# Investigation of genetic diversity of *Salmonella enterica* strains isolated from patients by Pulsed Field Gel Electrophoresis

**Published:** 2011-01-10

**Authors:** Mohsen Ghafari, Bita Bakhshi, Mohamad Reza Pour Shafi, Nour Amir Mozafari, Mona Salimi, Fateme Roosta

**Affiliations:** 1 Science & Resaerch Campus, Islamic Azad University, Tehran, Iran; 2 Department of Microbiology, School of Medical Sciences, Tarbiat Modares University, Tehran, Iran; 3 Microbiology Unit, Pasteur Institute of Iran, Tehran, Iran; 4 Department of Microbiology, Iran Medical Sciences University, Tehran, Iran; 5 Department of Biotechnology, Pasteur Institute of Karj, Tehran, Iran; 6 Science & Resaerch Campus, Islamic Azad University, Tehran, Iran

## 

Salmonellosis is one of the world's most common zoonotic infections that cause a wide spectrum of different diseases in organisms. *Salmonella* infection in humans maybe occurs as acute enterocolitis, intestinal fever (typhoid or Paratyphoid) and bacteremia with or without clinical signs. There must be differences between *Salmonella* enterocolitis and all cases of acute diarrhea, including invasive bacteria such as *Campylobacter jejuni*, *Shigella* species, invasive *E. coli*, *Yersinia enterocolitica*, etc. There is a variety of microorganisms with different biochemical and serological characteristics in Salmonella's gender. These organisms are infective in many animals, in addition to humans. They can attack to the foreign tissue of the intestine and can cause intestinal fevers. Typhoid fever is the most important infections among the outside bowel disease. In epidemiological studies, genotypic methods such as PFGE can help us in distinguish correlation between strains within a species, discovering the source of infection and its prevalence ways and finally lead us to control the infections. Among the genotypic methods, PFGE method called the gold standard method, but it is not applicable in all laboratories due to the complexity and high cost of equipment and materials normally required.

PFGE is a golden standard for typing of many of bacteria such as *salmonella enterica* species. This method has advantages such as universality (all bacteria can be typed by this method), high ability of repetition and high differentiation power. Due to the occurrence of diarrheal disease in passengers and the occurrence of epidemics in various countries, especially in developing and developed countries, (The National Molecular Sub typing Network for Food borne Disease Surveillance) PULSE NET, provided standard protocols for typing of strains of bacteria that based on genotyping with Pulsed Field Gel Electrophoresis. We can determine the genetic relationship between strains of bacteria with this technique and let us to attempt to provide an appropriate treatment with knowing genetic variation based on the mutation and determine the antimicrobial resistance factors. With this technique we can also determine the genetic relationship between strains isolated from patients with isolates from other countries and internal strains that have been caused epidemics and Andmys. We studied the genetic relationship, genetic development, genetic diversity, antibiotic resistance and clonal relationship of *Salmonella* strains isolated from patients using PFGE alongside other techniques such as determination of antibiotic susceptibility profile and attempted to explore their the epidemiological importance and antibiotic susceptibility.

